# Genomic signatures among *Oncorhynchus nerka* ecotypes to inform conservation and management of endangered Sockeye Salmon

**DOI:** 10.1111/eva.12412

**Published:** 2016-10-21

**Authors:** Krista M. Nichols, Christine C. Kozfkay, Shawn R. Narum

**Affiliations:** ^1^ Conservation Biology DivisionNorthwest Fisheries Science CenterNational Marine Fisheries Service, NOAASeattleWAUSA; ^2^ Idaho Department of Fish and GameEagleIDUSA; ^3^ Columbia River Intertribal Fish Commission, Hagerman Fish Culture Experiment StationHagermanIDUSA

**Keywords:** migration, natural selection, random forest, residency

## Abstract

Conservation of life history variation is an important consideration for many species with trade‐offs in migratory characteristics. Many salmonid species exhibit both resident and migratory strategies that capitalize on benefits in freshwater and marine environments. In this study, we investigated genomic signatures for migratory life history in collections of resident and anadromous *Oncorhynchus nerka* (Kokanee and Sockeye Salmon, respectively) from two lake systems, using ~2,600 SNPs from restriction‐site‐associated DNA sequencing (RAD‐seq). Differing demographic histories were evident in the two systems where one pair was significantly differentiated (Redfish Lake, *F*_ST_ = 0.091 [95% confidence interval: 0.087 to 0.095]) but the other pair was not (Alturas Lake, *F*_ST_ = −0.007 [−0.008 to −0.006]). Outlier and association analyses identified several candidate markers in each population pair, but there was limited evidence for parallel signatures of genomic variation associated with migration. Despite lack of evidence for consistent markers associated with migratory life history in this species, candidate markers were mapped to functional genes and provide evidence for adaptive genetic variation within each lake system. Life history variation has been maintained in these nearly extirpated populations of *O. nerka,* and conservation efforts to preserve this diversity are important for long‐term resiliency of this species.

## 1 Introduction

The current age of genomics provides the opportunity to investigate a more complete picture of genetic variation in natural populations that includes signatures of adaptive variation in addition to evolutionary history and connectivity (Luikart, England, Tallmon, Jordan, & Taberlet, 2003). In turn, understanding the processes that have led to extant diversity and differentiation among populations and adapted ecotypes is an important consideration for restoration, conservation, and management of previously extirpated, exploited, and threatened populations (Allendorf, Hohenlohe, & Luikart, 2010; Funk, McKay, Hohenlohe, & Allendorf, 2012; McMahon, Teeling, & Hoglund, 2014). As Shafer et al. (2015) aptly point out, there are still many uncertainties in how genomic information can be used broadly in a conservation framework, particularly for nonmodel species. However, genomic information can address questions regarding patterns of extant genetic diversity, how those patterns relate to environmental variables (Bragg, Supple, Andrew, & Borevitz, 2015), and whether or not potential adaptive genetic variation can be identified among variable ecological life history forms (Stapley et al., 2010).

Salmonid fishes native to streams draining into the north Pacific Ocean vary in timing and propensity for ocean migration as juveniles and timing of return migration for spawning in their respective natal streams (Groot & Margolis, 1991). Many species display both migratory (anadromous) and resident life histories, but differences in migratory tendency and timing vary within and among species. Importantly, these life history characters are often used as criteria for conservation and management of populations. Previous studies have suggested that heritable variation for migration and seawater adaptation exists within multiple salmonid species (see Carlson and Seamons (2008)) for a review), including variation between anadromous sockeye and resident Kokanee Salmon (*Oncorhynchus nerka*; Foote, Wood, Clarke, & Blackburn, 1992). Moreover, genetic correlations for characters related to migration vs. residency in rainbow and steelhead trout (*Oncorhynchus mykiss*) suggest that there is a genetic trade‐off between migration and residency (Hecht, Hard, Thrower, & Nichols, 2015). More recently, genomic studies have sought to evaluate molecular divergence between alternative resident and migratory ecotypes within salmonid species in an attempt to characterize the underlying genetic basis for migration. For example, several studies in *O. mykiss* have examined the genetic basis of migration (Hale, Thrower, Berntson, Miller, & Nichols, 2013; Hecht, Campbell, Holecek, & Narum, 2013) or “smoltification,” the physiological process that prepares juveniles for migration (Hecht, Thrower, Hale, Miller, & Nichols, 2012; Nichols, Felip, Wheeler, & Thorgaard, 2008). Limited parallel genomic divergence has been observed between migratory and resident forms within species (i.e., Pearse, Miller, Abadia‐Cardoso, & Garza, 2014), but the question of whether the same or different genetic mechanisms for other species that vary in migration and residency is largely unexplored.

In this study, we examine genetic differentiation among *O. nerka* populations that differ markedly in life history, to inform restoration and recovery actions for this protected species. Life history variation, within this and other species, is important in defining the discreteness or unique properties of populations considered for conservation and management policies. Four major life history variants have been described for *O. nerka* and are mainly delineated based upon the degree of juvenile freshwater residency. There are two anadromous, or migratory, life history types, whereby juveniles, born in freshwater, migrate to sea before returning to their natal streams to spawn. The first, “anadromous lake‐type,” is the most common rearing in lakes for ~1 year before migrating to the ocean where they spend ~1–2 years before returning to freshwater to spawn and die (Burgner, 1991). Within this type, there are also creek and beach spawners (Wood, 1995). The second, “anadromous sea‐or river type,” spends shorter amounts of time (weeks to months) in freshwater before migrating to the ocean and spawn in rivers rather than lakes (Wood, Bickham, Nelson, Foote, & Patton, 2008). Two life history types are resident, spending their entire lives in freshwater. “Resident Kokanee” spend their entire life in freshwater and are the progeny of freshwater parents (Nelson, 1968). An intermediate variant between the anadromous and resident forms, “residual” Sockeye Salmon, has also been documented (Ricker 1940, 1959). Residual Sockeye Salmon can be the progeny of anadromous or residual parents (P. Kline, IDFG, unpublished results), but remain in freshwater lakes to rear and reproduce rather than migrate to the sea. The distribution and prevalence of each life history variant likely depend upon available habitat, freshwater productivity (McGurk, 2000), proximity to the ocean, and difficulty of migration (Hendry, Bohlin, Jonsson, & Berg, 2004; Wood, 1995). Multiple forms can be found sympatrically within systems, and many display genetic divergence based upon strong fidelity to spawning areas, differences in spawn timing, and spawning locations (Lin, Ziegler, Quinn, & Hauser, 2008; McGlauflin et al., 2011; Quinn, Stewart, & Boatright, 2006). Genetic characterizations of sympatric forms of Kokanee and Sockeye Salmon have found that these two life history variants are generally more genetically similar to one another than each form is to the same form in neighboring lakes (Foote, Wood, & Withler, 1989; Taylor, Foote, & Wood, 1996; Wood & Foote, 1996). However, little documentation exists regarding the genetic relationship of residual fish to other sympatric forms given their elusive nature (although see [Cummings, Brannon, Adams, & Thorgaard, 1997; Waples, Aebersold, & Winans, 2011]).

Although *O. nerka* as a species is not at risk for extinction, there are a number of isolated populations throughout the range that are locally extinct or threatened (Rand et al., 2012). Including these are endangered Snake River Sockeye Salmon in central Idaho in the Salmon River Basin, wherein native anadromous Sockeye Salmon undertake some of the longest migrations known for the species. Until the early to middle part of the 20th century, anadromous Snake River Sockeye Salmon populations were abundant and supported by a number of lake systems in the upper Snake and Salmon River watersheds in northeastern Oregon and central Idaho. These included populations returning to the Wallowa, Payette, and the South Fork and upper Salmon rivers. To date, all but a single anadromous population found in Redfish Lake, on the upper Salmon River, are considered extirpated due to historical human activities in the watershed. The decline in population numbers led to the 1991 listing of Snake River Sockeye Salmon, under the US Endangered Species Act (56 FR 58619 1991). At that time, a captive broodstock program was initiated from the 16 wild anadromous adults that returned to Redfish Lake as well as outmigrating smolts and residuals that were collected in the early 1990s (Kalinowski, Doornik, Kozfkay, & Waples, 2012; Kline & Flagg, 2014). Current restoration efforts utilize the hatchery program to re‐introduce Sockeye Salmon into their native nursery lakes in the upper Salmon River Basin (Kline & Flagg, 2014).

There are three *O. nerka* ecological life history types in the extant populations in the upper Salmon River. In Redfish Lake, lake‐type, anadromous Sockeye Salmon spawn on beach shoals within the lake in October, but the lake also supports two resident *O. nerka* ecotypes. A resident, early‐spawning Kokanee population exists in Fishhook Creek, spawning in August in the stream, and rearing in Redfish Lake the remainder of the time. This form has been shown to be genetically divergent from anadromous Sockeye Salmon in Redfish Lake due to differences in spawn timing and location (Waples et al., 2011). A second resident ecotype in Redfish Lake includes a residual form that has been shown to be genetically similar to the anadromous form (Cummings et al., 1997). The residual and anadromous forms of *O. nerka* in Redfish Lake are considered the same population, and both are protected under the Endangered Species Act (Waples et al., 2011). Extant populations of resident Kokanee in the upper Salmon River Basin are found in Alturas Lake, Pettit Lake, and Stanley Lake although the latter two lakes have been impacted by varying degrees by prior hatchery releases of non‐native Kokanee (Bowler, 1990; Waples et al., 2011). While the Alturas Lake population is mainly characterized as a resident, early‐creek spawning Kokanee population, outmigrating smolts have been captured at a juvenile trap at the outlet of the lake and a small number of ocean‐returning adults have been documented since 2000, indicating that the propensity for migration exists within this population (National Marine Fisheries Service 2015). Understanding the genetic relationships of resident and anadromous *O. nerka* in Alturas Lake relative to the life history forms present in Redfish Lake populations is of particular interest, as recovery efforts focus on rebuilding locally adapted populations and conserving extant genetic and life history diversity in *O. nerka* in the upper Salmon River lakes.

In this study, we used markers generated from restriction‐site‐associated DNA sequencing (RAD‐seq) across the genome to evaluate signatures of demographic and evolutionary processes in *O. nerka* in the upper Salmon River Basin, Idaho. The objectives of this study were to test whether (i) population structure exists among four *O. nerka* collections of conservation concern in Redfish Lake and Alturas Lake, (ii) genomic loci show patterns of diversity consistent with adaptive divergence between migratory and resident life histories, (iii) particular regions of the genome are associated with life history diversity in these population pairs, and (iv) regions showing adaptive divergence or associations with life history are the same or different between the two population pairs which have markedly different demographic histories. Our findings illuminate the degree that genetic parallelism for divergence between migration and residency exists among these *O. nerka* populations, but most importantly provide invaluable genetic information that will assist with developing recovery strategies for endangered upper Salmon River Sockeye Salmon populations. This study highlights that contrasting patterns of genomic differentiation between pairs of migratory and resident life histories are an important consideration in defining conservation units and developing re‐introduction strategies to restore and recover naturally, spawning populations of Sockeye Salmon.

## 2 Methods

### 2.1 Sample collection

**Figure 1:**
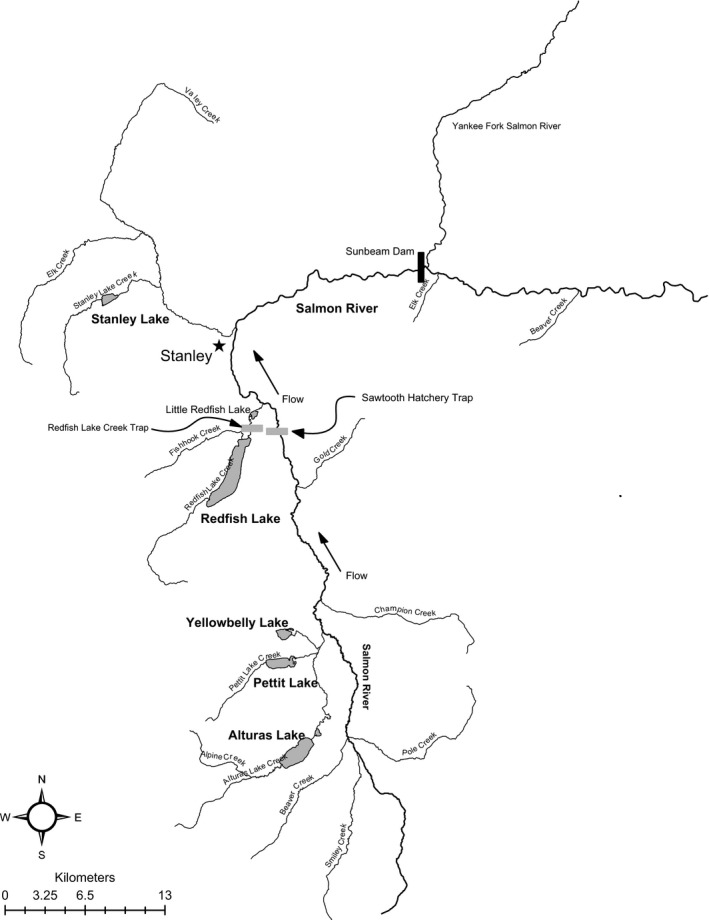
Map of study area (part of the upper Salmon River Basin) and sampling locations

Four collections of anadromous and resident *O. nerka* were obtained from two lake systems in central Idaho, in the upper Salmon River Basin (Fig. 1; Table 1). Sockeye Salmon from these lakes represent the only extant anadromous fish in the upper Snake River Basin (into which the Salmon River flows). In August 2012, 50 individuals were sampled from the resident Kokanee population in Fishhook Creek, a tributary to Redfish Lake near the outlet of the lake on the north side. Sampling was conducted by beach seining during spawning in Fishhook Creek. These Kokanee from Fishhook Creek will be hereafter referred to as “Redfish—Resident.” It is important to note that Redfish Lake also supports a residual population of *O. nerka* that remains entirely in freshwater, but genetic samples of those fish were not available for this study due to the difficulty in capturing and collecting samples from these fish. Anadromous Sockeye Salmon used in this study from Redfish Lake (“Redfish—Anadromous”) were collected from July through September in 2011 and 2012; 108 individuals were subsampled from all returning anadromous adults upon trapping at Redfish Lake Creek (“Redfish—Anadromous”). In Alturas Lake Creek, 50 resident Kokanee Salmon were sampled by beach seining during spawning in August 2012 (“Alturas—Resident”). Additionally, 19 individuals that collectively returned to the Redfish Lake Creek and Sawtooth weirs (see Fig. 1) from years 2008 through 2010 as anadromous adults from the Alturas Lake population (“Alturas—Anadromous”) were detected with genetic analyses and included in this study (Appendix S1). These 19 individuals that were intercepted at the weirs and assigned to Alturas Lake were the entirety of anadromous fish returning to that system in those years (National Marine Fisheries Service 2015). Beyond this study, only one additional anadromous individual has been identified, for a total of 20 returning anadromous adults with Alturas Lake origin since 1991 (C. Kozfkay, unpublished data). While stocking of non‐native Kokanee has historically taken place in both Fishhook Creek and Alturas Lake, the extant resident Kokanee in each of these streams are considered to be native populations based on genetic analyses by Waples et al. (2011). At the time of sampling, the length and sex of each individual were recorded and a fin clip was taken and stored in 95% ethanol for genetic analyses. DNA from all samples was extracted from fin tissue using the Qiagen DNeasy kit, following manufacturer's instructions (Qiagen, Valencia, CA), and used for genetic analyses described below.

**Table 1:** Sample sizes of collections in this study

Site	Sample sizes		
	Collected	Population genomics	GWAS
Alturas—Anadromous	19	14	14
Alturas—Resident	50	49	49
Redfish—Anadromous	108	105	103
Redfish—Resident	49	45	45
Total	228	213	211

### 2.2 RAD‐seq genotyping

Samples were prepared for restriction‐site‐associated DNA sequencing (RAD‐seq) as described by Miller, Dunham, Amores, Cresko, and Johnson (2007). Briefly, DNA was digested with *SbfI*, and then uniquely barcoded for library preparation. Barcoded samples were divided into three libraries containing 95, 95, and 38 individuals per library. To ensure adequate read depth, each library was sequenced for 100 cycles in two lanes of a single‐read flow cell on an Illumina HiSeq 1500, for a total of six lanes. Stacks v. 1.02 (Catchen, Hohenlohe, Bassham, Amores, & Cresko, 2013) was used to process the data. Reads produced from each lane of sequencing were first trimmed (to 75 bases), quality filtered, and demultiplexed according to sample barcodes. Unique stacks of possible alleles in each individual were grouped using ustacks, using a bounded SNP model that bounded sequencing error between 0.001 and 0.01, and all other parameters at the default value, except for the minimum depth of coverage to create a stack within an individual (−*m* parameter). For this parameter, a conservative value of −*m* 5 was used initially for individuals selected to construct the catalog from which SNP positions were identified and later genotyped. Twenty individuals, with total filtered reads between 2.5 and 3.5 million, were used to construct the catalog in cstacks, with default parameters except that −*n* was set to 2. Catalog individuals included five individuals from each of the four collections. Once the catalog was constructed, all individuals were processed again in ustacks prior to genotyping against the catalog, using −*m* 2 to allow alleles to be called, within the context of the maximum likelihood model for genotyping. All samples were aligned to the catalog using sstacks with default parameters. Individual genotypes were called as haplotype genotypes, for RAD tags possessing only two haplotypes. Genotypes were scored for each locus in each individual with a minimum depth of sequencing of 10 reads per locus (−*m* 10 in populations). Setting a minimum depth of sequencing per allele with −*m* 2 in ustacks for genotyping, together with a minimum depth per locus of −*m* 10 in populations balances the miscalling of heterozygotes as homozygotes in cases where some individuals have low sequence reads per locus. Individuals that had fewer than 75% of loci genotyped were removed from downstream analyses. Genotypes for retained individuals were further filtered to remove loci with (i) a global minor allele frequency (MAF) of less than 0.05, (ii) greater than 80% observed heterozygosity to remove likely paralogous sequence variants (PSVs), (iii) greater than 30% of individuals with missing data, and (iv) removing loci that were consistently out of Hardy–Weinberg equilibrium (HWE) in three or more collections. Most PSVs will have been removed by excess heterozygosity and HWE filters. However, without a genome or a curated set of loci of known PSVs in this species and these populations, it cannot be known how many PSVs were scored as disomic loci and retained in our analyses. A 5% false discovery rate, using the Benjamini and Yekutieli (2001) method (hereafter referred to as the BY‐FDR), was used for tests for significant departures from HWE and for all other significance tests described below, as it provides a balance between type I and type II errors (Narum, 2006). Filtering steps were conducted using a combination of a SQL database built from Stacks output, populations in Stacks, and vcftools (Danecek et al., 2011). Hardy–Weinberg exact tests were implemented for each locus and each population using Genepop v. 4.0 (Rousset, 2008). The final filtered set of loci (see Appendix S2 for genotypes) was used for annotation, population genomics, and association genetics.

### 2.3 Genome alignment and annotation of RAD loci

RAD loci passing filtering and used for statistical analyses were aligned to two previously published linkage maps for Sockeye Salmon (Everett, Miller, & Seeb, 2012; Limborg, Waples, Allendorf, & Seeb, 2015), to the Rainbow Trout (*Oncorhynchus mykiss;* Berthelot et al. (2014)) and Atlantic Salmon genomes (*Salmo salar* (Davidson et al., 2010; Lien et al., 2016), accession AGKD00000000.3 for scaffolds and ICSASG_v2, GCF_000233375.1 for assembled chromosomes), and to *O. nerka* contigs identified from cDNA sequencing of beach and stream spawning Kokanee Salmon (Lemay, Donnelly, & Russello, 2013; Lemay & Russello, 2015). The goal was (i) to evaluate the genomic position of RAD loci in order to better compare genome regions of interest among populations, studies, and species and (ii) to provide annotation for regions linked to RAD loci. Alignment to the Sockeye Salmon linkage maps was conducted by aligning RAD loci from this study to that of Everett et al. (2012) and Limborg et al. (2015) with bowtie, allowing three mismatches and reporting only the best and unique match. RAD loci from this study were trimmed to 60 bases to conform to the shorter lengths used in Everett et al. (2012). Alignment of the *O. nerka* RAD loci to Rainbow Trout and Atlantic Salmon genomes and *O. nerka* contigs was conducted using bowtie2, using the default parameters and reporting the best match (Langmead & Salzberg, 2012). The Rainbow Trout assembly consists of scaffolds that are annotated with the locations of putative and known coding regions, which facilitated the identification of genes linked to SNPs in this study. Some but not all Rainbow Trout scaffolds have been assigned to chromosomes, which provides limited genome position information. In contrast, the Atlantic Salmon genome currently consists of full‐length chromosome sequences (Lien et al., 2016). Two alignments were performed to the Atlantic Salmon genome: (i) to scaffolds, as it is expected that markers in close linkage on scaffolds are expected to be conserved in closely linked blocks across the two species and (ii) to the newer annotated Atlantic Salmon genome assembly presented as contiguous chromosomes, to provide information on synteny among *O. nerka* and *S. salar* chromosomes. Alignment to the shorter scaffold segments facilitated incorporating markers found on the same scaffolds into *O. nerka* linkage groups, on the basis of markers from this study that were anchored on the *O. nerka* linkage groups. Only RAD loci from this study that mapped to a single genome location were used for further annotation. Any coding sequence within 5 kb of the mapped RAD loci was used for gene annotation. Annotation of CDS from *O. mykiss* was accomplished by blastx to the complete human and zebrafish ENSEMBL peptide and coding sequence databases (release 78, December 2014). Annotation of linked *S. salar* genes was taken from the NCBI annotation of mRNA within 5 kb of mapped *O. nerka* RAD loci. Finally, linkage disequilibrium (LD) was evaluated between markers identified for divergent natural selection between life histories and in association analyses, using the R package LDheatmap (Shin, Blay, McNeney, & Graham, 2006).

### 2.4 Population genomic analyses

To test the hypotheses that individual loci in the genome showed patterns of divergence consistent with the actions of divergent natural selection between life history forms, we used the empirical method employed by LOSITAN (Antao, Lopes, Lopes, Beja‐Pereira, & Luikart, 2008). This approach, built on FDIST2 (Beaumont & Nichols, 1996), has been shown to have relatively low type I and type II errors in the detection of loci under divergent natural selection under evolutionary scenarios such as those observed in this study system (Lotterhos & Whitlock, 2014; Narum & Hess, 2011). To test for outliers, three different datasets were evaluated comparing (i) the Kokanee from Fishhook Creek with the Sockeye Salmon from Redfish Lake (Redfish—Resident vs. Redfish—Anadromous), (ii) resident and anadromous *O. nerka* from Alturas Lake (Alturas—Resident vs. Alturas—Anadromous), and (iii) all four populations considered together. Analyses between pairs of life histories within each lake were expected to test for loci underlying selection for migratory characteristics in each system. The analysis of all four populations combined was used to identify, together with pairwise analyses, loci that were putatively neutral for population genetic analyses. Significance of loci showing divergent or stabilizing natural selection was evaluated with corrections for multiple tests using a BY‐FDR. A set of putatively neutral loci was devised from the LOSITAN results from the three analyses, such that each locus contributing to the neutral set was found to have empirical *p* values from the LOSITAN analyses between 0.1 and 0.9 in all three analyses. The neutral and selected sets of loci were then used to evaluate population genetic variation and structure in several ways. Adegenet (Jombart, 2008) was used to evaluate axes of genetic variation within and between populations using principal component analyses in the full, neutral, and selected sets of loci. The neutral set of loci was used to evaluate the potential numbers of populations (or clusters) represented from the four collections of samples in this study, using fastStructure (Raj, Stephens, & Pritchard, 2014). FastStructure was run using default parameters, a logistic prior, and K from 2 to 4, for 100 replicate runs at each K. Genepop v. 4 (Rousset, 2008) was used to estimate diversity statistics including observed and expected heterozygosity, and the fixation index. The R package StAMPP (Pembleton, Cogan, & Forster, 2013) was used to estimate unbiased *F*_ST_ (Weir & Cockerham, 1984) between pairs of populations, as well as *p*‐values and 95% confidence intervals (CI) of *F*_ST_ based on bootstrap (*n* = 1,000) sampling. Finally, effective population size was evaluated using the linkage disequilibrium method (Waples & England, 2011) with the neutral SNP loci using the program NeEstimator v. 2 (Do et al., 2014).

### 2.5 Genomewide association analyses

Two modeling approaches were taken for genomewide association analyses (GWAS) to test for associations between genotype and life history. These approaches were (i) a single locus‐by‐locus approach in a traditional framework for GWAS and (ii) a multiple locus approach that simultaneously accounts for the contribution of multiple loci to phenotypic variation, using a random forest model. In both association analyses, life history was encoded as a binary trait, whereby migratory individuals were scored as ones, and resident Kokanee individuals were scored as zeros. Both methods benefit from complete genotype data on all individuals (and in the case of random forest, will not allow missing data). Missing genotypes were therefore imputed using fastPHASE 1.4.0 (Scheet & Stephens, 2006). Three subpopulations were coded in fastPhase as population genetic analyses suggested no substructure between resident and anadromous collections from Alturas Lake. Population structure and co‐ancestry was accounted for in both approaches to control for false genotype–life history associations that may arise due to these effects, as described below.

#### 2.5.1 GWAS

GWAS was performed using the R program GAPIT (Lipka et al., 2012), which implements a linear mixed effects model (Zhang et al., 2010) testing each locus for association with migration or residency. In the linear mixed effect model, sex and principal component scores to account for underlying genetic structure were included as potential fixed effects, while kinship calculated from the data was included as a random effect (VanRaden, 2008). The fixed effects were evaluated using a model selection procedure for association with the binary life history type in GAPIT, and only significant terms remained in the model for genetic association tests. Three GWAS analyses were conducted to evaluate genetic associations with life history in (i) resident and anadromous *O. nerka* from Alturas Lake, (ii) resident and anadromous *O. nerka* from Redfish Lake, and (iii) all four collections together. The fit of the resulting model to the data was evaluated with QQ plots, and significance of loci was evaluated with corrections for multiple tests using a BY‐FDR.

#### 2.5.2 Random forest

In contrast to GWAS, the random forest analysis accounts for the effects of all or many loci simultaneously, evaluating effects on the inclusion of individual markers on the proportion of total phenotypic variation explained. Random forest was implemented on the same three datasets (Alturas only, Redfish only, and a combined analysis), as was carried out for GWAS above. To account for population structure, possible familial relationships, and sex in the random forest analysis, genotype, and phenotype data were corrected for these factors prior to random forest analysis (Brieuc, Ono, Drinan, & Naish, 2015; Zhao et al., 2012). To calculate residual phenotypes and genotypes for each of these datasets, the vanRaden kinship matrix and significant principal component scores for population structure, estimated in GAPIT, along with sex, binary life history, and fast PHASE imputed SNP genotypes were imported into TASSEL 5.2.2 (Zhang et al., 2010). A mixed linear effects model (MLM) was implemented in TASSEL using sex and principal component scores as fixed effects, and the kinship matrix, as a random effect to model either (i) the binary life history phenotype, or (ii) each marker genotype which had been coded as 0, 1, or 2 for homozygous minor allele, heterozygotes, and homozygous major allele, respectively. From the MLM, residuals for the binary phenotype and residuals for numeric genotypes were output from TASSEL for random forest analysis. Random forest analyses were run using the R package randomForest (Liaw & Wiener, 2002), with initial triplicate runs evaluating 10,000 regression trees with all markers. To determine whether the three independent runs of the random forest had converged on the same relative importance values for markers, Pearson correlation coefficients were calculated between marker importance values. If the correlation coefficients were less than 0.90, the random forest was rerun in triplicate for 100,000 trees. With the triplicate random forest runs, the markers were ranked in the order of their relative importance values in the full model, and top subset of markers was selected to rerun random forest analyses to evaluate the proportion of variation explained by each subset. The proportion of variance explained was taken as the pseudo‐*R* ^2^ value returned by the random forest regression model at the last iteration. Finally, once it was determined the range of total markers for which the greatest amount of variation was explained, a backwards purging approach was implemented to determine the final number and identity of the best subset of markers that explained the greatest total variation in the corrected phenotypes, as described by Holliday, Wang, and Aitken (2012) and Brieuc et al. (2015). The backwards purging approach included running the random forest analysis again on the subset of markers chosen, purging the single marker with the lowest relative importance value in the model, until all but two markers remain in the model. In all cases, from the original random forest analyses using all markers, to analyses including runs of the top subsets and backwards purging, three random forest analyses were run with at least 10,000 trees; a greater number of trees was run to achieve Pearson correlations greater than 0.90 between marker importance values in three independent random forest analyses. Metrics used to evaluate the proportion of variation explained were taken as the average pseudo‐*R* ^2^ from the last iteration of each of the three random forest analyses run. A final model that included markers explaining the greatest proportion of variation in phenotype from the backwards purge approach was chosen as the best subset of markers that maximized the total proportion of variation in the corrected phenotype.

## 3 Results

### 3.1 RAD‐seq

The average number of quality‐filtered reads retained was approximately 3.7 million reads per individual (range 11,423 to 9,411,324; Table S1). Although 228 individuals were initially included, two individuals were removed from the Alturas—Anadromous collection prior to further analyses due to ambiguous population membership (Appendix S1). Another 10 individuals were removed from further analyses because > 25% of loci were missing genotype data. In total, 213 individuals were retained for population genomics analyses, and 211 were retained for GWAS (two individuals were missing sex information; Table 1). Filtering of loci for proportion genotyped, removal of PSVs, and removing loci with MAF < 0.05 resulted in 2,593 polymorphic RAD tags retained for statistical analyses and genome alignment and annotation (see Table S2 for filtered tag sequences). Of these tags, only 32 tags (1.2%) had three or greater SNPs per tag, 210 (8.1%) contained two SNPs per tag, and 2,351 tags (90.7%) contained only a single SNP per tag. Tags with more than one SNP were treated as haplotypes. With 2,593 polymorphic tags used for each statistical test, the empirical threshold for *p*‐values was *p* = 0.005926 for a 5% BY‐FDR.

### 3.2 Genome alignment and annotation of RAD loci

A summary of alignments and annotations to the sockeye linkage map and the Rainbow Trout and Atlantic Salmon genomes is provided in Table S3. RAD tags from this study matched to 401 and 568 loci from the *O. nerka* linkage maps of Everett et al. (2012) and Limborg et al. (2015), respectively; 123 (4.7% of all markers in this study) aligned RAD tags from this study were found in both of the linkage maps, with a total of 846 loci (32%) mapped to at least one of the *O. nerka* linkage maps. For alignment of RAD tags to the Rainbow Trout genome, 1866 RAD tags (72% of all loci) aligned to at least one position. Of those that aligned, 1028 RAD loci (39%) aligned to exactly one genome position, while another 846 loci (33%) aligned to more than a single position. Of the *O. nerka* RAD tags aligning uniquely to *O. mykiss*, 593 mapped to *O. mykiss* scaffolds with information on chromosome location. Only 50 mapped to known positions on chromosomes, while another 543 markers were assigned to *O. mykiss* scaffolds with chromosome information only (i.e., no positional information on the chromosome); the remaining 435 *O. nerka* RAD tags aligning to the *O. mykiss* genome were localized to scaffolds without chromosome information. RAD tags from *O. nerka* collections mapped to every *O. mykiss* chromosome except chromosome 25. There were 510 *O. mykiss* coding sequences (CDS) within 5 kb of the aligned sockeye RAD tags, with an average distance of 510 bases between the CDS and the starting matched position of the sockeye RAD tag. In the Atlantic Salmon genome, 1,452 (56%) of the *O. nerka* RAD tags mapped to at least one scaffold when using only the scaffold database, and of those aligned, 699 aligned to a single position. Of the 726 *O. nerka* RAD loci aligned to a unique position in the *S. salar* genome chromosomes, 502 loci were within 5 kb of a CDS; the average distance from the alignment start to the predicted CDS in the Atlantic Salmon genome was 268 bp. There were 754 (29%) aligned RAD tags that were identified to be within 5 kb of CDS in either the *S. salar* or *O. mykiss* genomes (or both). Summarizing the alignment of *O. nerka* RAD tags to the salmonid genome sequences, 52% of the sockeye RAD tags aligned to a single position in one or both of these salmonid genomes (22.2% of tags align to both genomes, 17.4% only to *O. mykiss*, and 12.5% only to *S. salar*). In total, 1704 RAD tags (66%) aligned to a single position in at least one of these resources (either of the genomes, or to the *O. nerka* linkage maps). There were a number of markers that did not align directly to the sockeye linkage maps, but alignment of *O. nerka* RAD markers to the Atlantic Salmon and Rainbow Trout genomes allowed the placement of additional markers to sockeye linkage groups. For example, if a single RAD marker from this study aligned to the linkage map, but additional markers aligned to the same Rainbow Trout or Atlantic Salmon scaffolds, it can be deduced that the markers are linked to one another in the *O. nerka* genome because many of the scaffolds from the other salmonid genomes are short fragments of physical chromosomes. With this information, we were able to localize an additional 190 RAD tags from this study to *O. nerka* linkage groups, for a total of 1036 RAD markers from this study (~40% of total) assigned to an *O. nerka* linkage group. Two markers from this study were also aligned to the 18 outlier loci identified as outlier loci between beach and stream spawning Kokanee in Okanagan Lake (Lemay & Russello, 2015). RAD tags 36561 and 37622 in this study matched to RAD tags 14305 and 33487 from Lemay and Russello (2015), respectively.

### 3.3 Population genomic analyses

#### 3.3.1 Outlier analysis

In the Alturas collections, 225 loci (8.7% of 2593 markers tested) were identified as outliers between anadromous and resident life histories, and 71 (2.7%) of those showed signatures of divergent natural selection. In Redfish Lake, comparing the Sockeye Salmon to the Kokanee of Fishhook Creek (Redfish—Anadromous vs. Redfish—Resident), 103 loci (4%) were identified as outliers, and all of those loci showed signatures of divergent natural selection. Only two loci showing patterns of divergent natural selection overlapped between the two pairs of populations (Table S3). The loci showing patterns of divergent natural selection in the population pairs were distributed across the genome, although notably, a larger concentration of candidate divergent loci in the Redfish—Anadromous versus Redfish—Resident comparison were localized to linkage groups 12 (*n* = 7 loci) and 20 (*n* = 9 loci) based on synteny with the sockeye linkage maps, and by proximate physical location based on location within the same *O. mykiss* or Atlantic Salmon scaffold (Table S3). Markers on linkage group 12 in Sockeye Salmon map to scaffolds found on Rainbow Trout chromosomes 9 and 29. Outlier loci on *O. nerka* linkage group 20 map to scaffolds found on Rainbow Trout chromosomes 16 and 28. In both linkage groups, outlier loci generally have a lower observed heterozygosity in the Redfish—Anadromous population when compared to the Redfish—Resident population (Fig. S1). A single outlier identified between life history types in Redfish Lake (RAD tag 37622, on *O. nerka* linkage group (i) matched to an outlier locus identified between beach and stream spawning Kokanee in Okanagan Lake (RAD locus 33487; [Lemay & Russello, 2015; ]); this locus has been annotated as decapentaplegic and Vg‐related 1 (*DVR1*) in both Lemay and Russello (2015) and Everett et al. (2012) and is found on linkage group 1 in the Sockeye Salmon map. *DVR1* is also known as growth differentiation factor 3 (*GDF3*), which was the annotation of RAD tag 37622 in Atlantic Salmon in this study. When all four collections were evaluated together to identify outliers and a putatively neutral set of loci, 128 candidate loci for natural selection were identified, and 47 of those showed evidence for divergent natural selection (unusually high levels of differentiation among populations). There were 1036 loci (40%) that satisfied our criterion for putatively neutral SNPs, with LOSITAN posterior probabilities between 0.1 and 0.9 in all three datasets analyzed. Hereafter referred to as “neutral loci,” these SNPs were further used to evaluate population genetic structure, with results below. Results from outlier analyses are in Table S4.

#### 3.3.2 Population differentiation & structure

**Figure 2:**
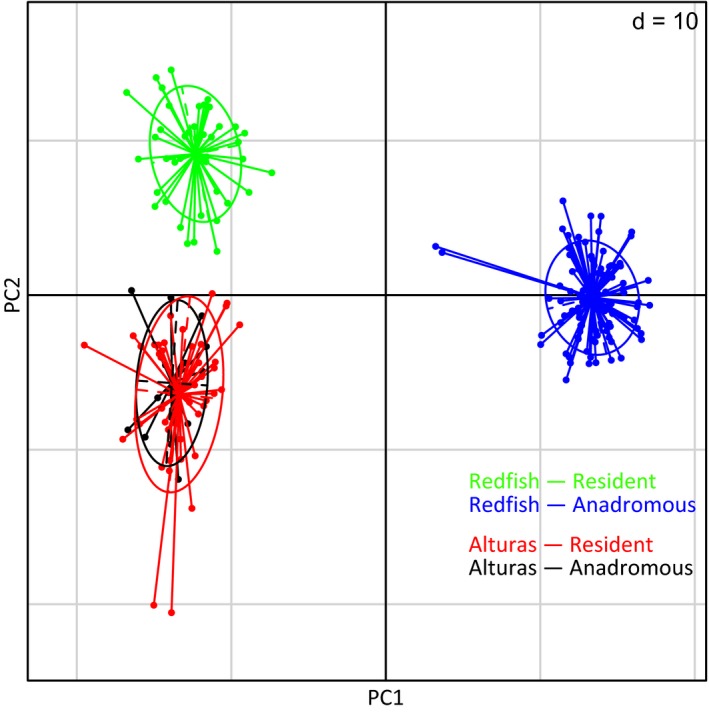
Axes of genetic variation with the neutral set of SNP loci (*n* = 1036). Ellipses represent the 95% inertia ellipse for collections represented in different colors

**Figure 3:**
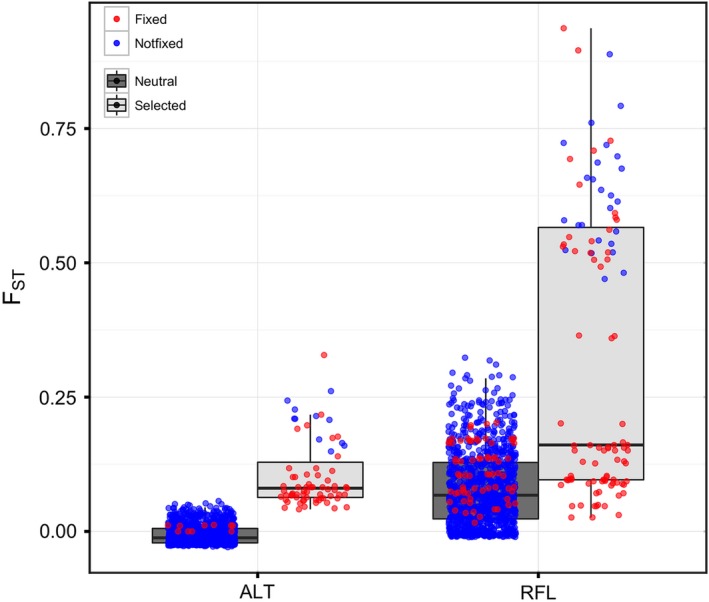
Boxplots for pairwise *F*_ST_ calculated from the neutral (*n* = 1036) and divergent selected loci in each population pair. *F*_ST_ values are those calculated in LOSITAN. Red and blue colors denote whether or not the locus was fixed in one of the two populations considered in each pairwise comparison

Results evaluating population structure and neutral genetic diversity largely suggested the presence of three randomly mating populations using the neutral set of loci (*n* = 1036): (i) Redfish—Anadromous, (ii) Redfish—Resident, and (iii) resident and anadromous *O. nerka* from Alturas Lake as a single population (Fig. 2 and Fig. S2 [fastStructure results]). No genetic differentiation was observed between Alturas—Anadromous and Alturas—Resident collections, except at a very small number of outlier loci. Observed and expected heterozygosity, and the fixation index are reported in Table 2. *F*_ST_ estimates between the four collections also supported this observation, with nonsignificant pairwise *F*_ST_ values between the Alturas—Anadromous and Alturas—Resident collections. The candidates for divergent natural selection identified within each of the population pairs showed very different results between the two systems (Fig. 3). In the Redfish—Anadromous and Redfish—Resident populations, much larger levels of differentiation were observed at outlier loci, and notably, all of those loci showed signatures of divergent natural selection. In contrast, lower levels of differentiation were observed between Alturas—Anadromous and Alturas—Resident individuals with fewer candidate loci identified for divergent natural selection. Estimates of contemporary effective population size (*N*_e_) using the neutral set of loci in the three populations identified from population genetic analyses ranged from 246 (95% confidence interval [CI]: 234–259) in Redfish Lake (Redfish—Anadromous), to 334 in the Alturas Lake (Alturas—Anadromous and Alturas—Resident combined; 95% CI: 308–366), and to 877 in Fishhook Creek (Redfish—Resident collection; 95% CI: 653–1329). It is expected that these estimates, although comparable between populations using the same sets of loci, were likely downwardly biased owing to both the inclusion of loci that are linked, and of mixed age, mixed cohort samples. Overall LD calculated in the estimation of N_e_ was 0.022 for the Alturas combined collection, 0.032 for the Redfish—Resident population, and 0.014 for the Redfish—Anadromous population.

**Table 2:** Mean (±SE) diversity statistics for the neutral set of SNP loci, including observed heterozygosity (*H*_o_), expected heterozygosity (*H*_e_), and the fixation index (*F*). “By population” indicates statistics where Alturas Lake and Alturas Lake Creek are combined for a single population

Collection	By collection			By population		
	*H*_o_	*H*_e_	*F*	*H*_o_	*H*_e_	*F*
Alturas—Anadromous	0.340 ± 0.006	0.316 ± 0.004	−0.054 ± 0.010	0.318 ± 0.005	0.323 ± 0.004	0.0307 ± 0.008
Alturas—Resident	0.311 ± 0.005	0.322 ± 0.004	0.0487 ± 0.008			
Redfish—Anadromous	0.280 ± 0.006	0.272 ± 0.005	−0.009 ± 0.007	0.280 ± 0.006	0.272 ± 0.005	−0.009 ± 0.007
Redfish—Resident	0.305 ± 0.005	0.317 ± 0.004	0.050 ± 0.008	0.305 ± 0.005	0.317 ± 0.004	0.050 ± 0.008

### 3.4 Association analyses

#### 3.4.1 GWAS

In the three subsets of data, different covariates were selected by model selection for inclusion in the MLM model. In the Alturas—Anadromous and Alturas—Resident pair, only sex was included, and in the Redfish—Anadromous and Redfish—Resident pair, the first two PC scores and sex were included. In the analysis of the combined collections, the first PC score and sex were included in the model. The model fit was best for the combined and Alturas only datasets (Fig. S3). A total of 54 unique loci (2.1% of total) were associated with migration/residency in this study (Table S5). In the analysis of resident and anadromous collections from Alturas Lake, only 10 loci (0.4%) were significant in the GWAS at an FDR of 0.05 while 27 loci were significant in the Redfish—Anadromous and Redfish—Resident pair. In the combined analysis of all four collections, 23 loci (0.9%) were associated with the migration/residency phenotype. There was greater overlap in the significant loci identified in the Alturas collections and the combined analysis, than either of those datasets overlapped with the analysis in the Redfish Lake—Fishhook Creek collections. A single locus was significant in all three datasets. This RAD locus, 16599, was not mapped to the existing Sockeye Salmon linkage maps, but was localized to Rainbow Trout chromosome 3 in an undetermined position, and to an Atlantic Salmon scaffold on chromosome 5 (Table S3). This SNP is within the boundaries of the start and stop codons of both the *O. mykiss* and *S. salar* coding sequences annotated as small nuclear ribonucleoprotein 48 kDa (U11/U12) (*SNRNP48*).

#### 3.4.2 Random forest

The same covariates significant from the GWAS models were used to calculate residual genotypes and phenotypes for each of the three datasets used for random forest. In the Alturas dataset, 100,000 trees were used in the random forest; in the combined and Redfish datasets, 10,000 trees were used. In all three datasets, ~2% or fewer markers maximized the total proportion of variation explained (Fig. S4). For the Alturas, Redfish, and combined analyses, an inclusive list of the top 2% of markers from each of the three initial random forest analyses included 28, 66, and 77 polymorphic RAD tags, respectively, which were used in the backwards purging approach. In the Alturas analysis, 11 loci (0.4% of all loci) explained the maximum of 56% of the variation in life history (Fig. S5); four of those loci were localized to four different *O. nerka* linkage groups, and the remaining loci were unaligned. In the Redfish Lake analysis, 24 loci (0.9%) explained the maximum of 58% of the total variation in life history (Fig. S5); 14 of those were localized to 11 different linkage groups, with multiple markers mapped to *O. nerka* linkage groups 4 and 12 (Table S3). In the combined analysis, 18 loci (0.7% of all loci) from the backwards purge maximized the proportion of variation (38.3%) explained for life history (Fig. S5). Of these 18 loci, seven were located to the sockeye linkage maps, spanning across six linkage groups (Table S3). Two of the loci (48300 and 47821) were located on the sockeye linkage group 4 (*r* ^2^ = 0.13), and one of these markers also mapped to an *O. mykiss* scaffold on chromosome 5.

### 3.5 Overlap in loci identified between populations and analyses

**Figure 4:**
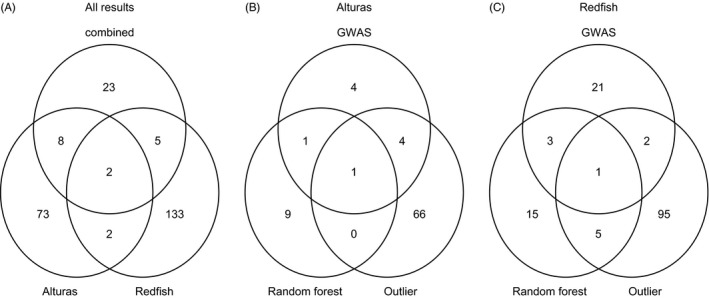
Venn diagrams of significant candidate loci identified in each of the datasets, combining loci identified from the outlier, GWAS, and random forest analyses (A), and in separate analyses of the Alturas (B) and Redfish (C) analyses

There was generally little overlap in the SNPs associated with divergence in life history between the two pairs of populations (Fig. 4A, Table S3). Four loci (0.1% of loci) were identified in both the Redfish and Alturas collections in at least one analysis. RAD tag 74226 was identified as an *F*_ST_ outlier in pairwise comparisons between life histories in both lakes, and in the Alturas only and combined GWAS. This locus was found on the same linkage group (*O. nerka* linkage group 23), homologous Rainbow Trout chromosome (chromosome 16), and the same Atlantic Salmon scaffold (ccf1000000006_0‐0, chromosome 19) as other markers identified as outliers or for associations with life history in the Alturas—Anadromous vs. Alturas—Resident contrasts. This RAD tag was located between the start and stop codons of coding regions in both *O. mykiss* and *S. salar*, but the annotations of those genes were discordant (see Table S3). RAD tag 16599 was not localized to a sockeye linkage group, but was found to be within the start and stop codons of the U11/U12 small nuclear ribonucleoprotein 48‐kDa protein‐like gene (*SNRNP48*) in both the Rainbow Trout and Atlantic Salmon genomes. The remaining two RAD tags were not located to any genome resource.

There was also little overlap in loci identified from the three different analyses (outlier analysis, GWAS, and random forest analyses) (Fig. 4B,C, Table S3). Although there was little overlap in the actual SNPs identified from each statistical approach, aligning the loci to available linkage maps and genome sequences provided a more integrated picture of regions associated with life history divergence. In Alturas, there were six loci identified in at least two analyses; these loci were located on three *O. nerka* linkage groups (7, 12, and 23), with three loci located to linkage group 12, and another locus unassigned to a linkage group. Only one locus, RAD tag 18540, was found to be within 5 kb of a gene (neuroligin‐2‐like gene, located on *S. salar* chromosome 18). As mentioned above, a number of loci were identified on *O. nerka* linkage group 12, which is the same linkage group as seven additional loci that were identified as candidates for divergent selection in the Redfish—Anadromous vs. Redfish—Resident comparison. In the Redfish dataset, there were 11 loci identified in at least two analyses. These loci were found on five different *O. nerka* linkage groups (3, 12, 20, 25, and 27); two loci were on linkage group 12, and five loci were unassigned to linkage groups. Three of these loci were found to be within 5 kb of genes (and all three were within the boundaries of start and stop codons) and include atrial natriuretic peptide receptor 1‐like (RAD tag 84702), leucine‐rich repeat‐containing protein 9‐like (RAD tag 57884), and integrin beta‐3‐like (RAD tag 74226) genes.

In total, there were 246 RAD loci (9.5% of total) that were significant in at least one analysis, which included all pairwise analyses and the combined analyses for GWAS and random forest. Linkage disequilibrium between all significant markers indicated the r^2^ between these markers was generally low (*r* ^2^ < 0.2; Table S6). There were some cases of pairs or clusters of markers with higher LD (Fig. S6); 84 markers in the significant set were linked (*r* ^2^ < 0.2) to at least one other marker. However, LD analysis suggests that the loci identified in the different tests are not in fact closely linked to one another, for the most part. This appears to indicate there are different loci in the population pairs contributing to variation in migration and residency, and different loci showing signatures of divergence between those pairs.

## 4 Discussion

Understanding the genetic basis of adaptive phenotypic divergence in populations is an important component in understanding the evolution of life history variation, and in consideration of conservation and management objectives in protected species. In this study, we did not find compelling evidence for parallel adaptive genetic divergence between two pairs of anadromous and resident *O. nerka* collections based on ~2,600 SNP markers distributed across the genome. A few regions of the genome overlapped in the identification of markers under selection, or associated with life history between the Redfish and Alturas pairs, but many loci were found in only a single analysis or population pair. In Redfish Lake, marked population differentiation exists between Sockeye Salmon and resident Kokanee sampled; however, in neighboring Alturas Lake, anadromous Sockeye Salmon are genetically similar to resident Kokanee, suggesting that the resident population produces a small number of outmigrating individuals that return to the system as adults. These findings will have an immediate impact on recovery and restoration for *O. nerka* in the upper Salmon River Basin. Moreover, and more generally, our findings demonstrate that differences in the genetic differentiation of ecological life history variants are an important consideration in conservation, recovery, and restoration efforts.

### 4.1 Genetic differentiation between migratory ecotypes

Studies in genetic parallelism of adaptive divergence are numerous in nonmodel animal species, but the evidence for shared mechanisms for diversification at the genomic level is presently limited. For example, several studies in stickleback show that the gene, ectodysplasin, consistently underlies adaptive differentiation between marine and freshwater populations (Colosimo et al., 2005; Hohenlohe et al., 2010; Jones et al., 2012), with several additional regions identified depending on population (e.g., Jones et al. (2012)). In Atlantic Salmon, a single major gene (*VGLL3*) is associated with age at sexual maturation (Ayllon et al., 2015; Barson et al., 2015). Many studies have found little to no parallelism in the genetic regions associated with life history divergence within and across populations and species (Roesti, Hendry, Salzburger, & Berner, 2012; Westram et al., 2014), which is consistent with the findings herein. Studies in salmonids have shown that there are multiple regions associated with life history differentiation, but few have found that the same regions in the genome were consistently associated with divergence between migratory and resident pairs (Hale et al., 2013; Hecht et al., 2012, 2013; Limborg et al., 2012; Narum et al., 2011). One notable exception is the identification of a single genetic region in *O. mykiss* on chromosome 5, in pairs of above and below barrier resident and migratory populations, respectively, from northern California and southern Oregon (Pearse et al., 2014). In the study herein, we did not observe a significant and consistent signal on *O. nerka* linkage groups that are homologous to *O. mykiss* chromosome 5, with the caveat that only ~23% of all markers in this study were located to *O. mykiss* chromosomes, and many of those lack positional information within chromosomes. In *O. nerka*, prior studies have shown that beach and stream spawning Kokanee in different systems lack evidence for parallel divergence at the genetic level for these differences in life histories (Frazer & Russello, 2013; Lemay & Russello, 2015). In our study, we also find little overlap with loci identified as outliers between those identified previously (Frazer & Russello, 2013; Lemay & Russello, 2015) However, a single locus identified between beach and stream Kokanee in Lemay and Russello (2015), *DVR1*, was identified as an outlier in Redfish Lake in this study. There are a number of possible reasons this and other studies fail to find common genetic architectures for parallel phenotypic divergence. Alternative genetic pathways could exist due to different colonization histories and subsequent adaptation across heterogeneous environments. Phenotypic plasticity could also play a contrasting role across landscapes. And finally, even as genomics has empowered studies in nonmodel species, incomplete sampling of the genome across individuals could limit the power to detect parallelism.

If migration and residency have evolved from standing genetic variation within parallel systems, and by the same underlying genetic mechanism, one may expect to find the same regions associated with this polyphenism across populations. However, one extremely important caveat to ecological genomic studies is that, although we are far more capable of surveying large portions of the genome in nonmodel species, marker density is still limited in answering questions regarding parallel adaptive divergence, particularly for traits that are expected to have a complex, polygenic architecture. Migration and dispersal are key components of life history evolution across the animal kingdom, but understanding how these life histories are shaped across landscapes is still limited by the availability of comprehensive, complete genome resources (Liedvogel, Akesson, & Bensch, 2011). Asking questions about parallel genetic divergence is further complicated by variable demographic histories, selection, and the extent of linkage disequilibrium across the genome, which is as yet largely unexplored in salmonid genomes; each of these factors are important considerations for questions of the genes underlying parallel divergence in complex traits (Haasl & Payseur, 2015). In monarch butterflies (*Danaus plexippus*), for example, the availability of a genome sequence, together with broad, global sampling of migratory and resident populations, has enabled a more complete reconstruction of migratory life history evolution, pointing to divergence from an ancestral migratory population in North America, and divergence in a few major genes (Zhan et al., 2014). In Swainson's thrushes (*Catharus ustulatus*), previous studies had shown a limited number of candidate genes associated with migratory life history, but analyses of genetic differentiation within the context of a sequenced genome provided a more complete evolutionary history of this complex. Delmore et al. (2015) have further demonstrated that genes associated with migratory traits were localized to islands of differentiation within the genome. As complete genome sequences become available for more salmonid species, and in particular for *O. nerka*, additional and more extensive future analyses will benefit from an improved genomic framework (see Vatsiou, Bazin, and Gaggiotti (2015), Manel et al. (2016) for review) under which to detect signatures of natural selection among migratory and resident types.

Although there was little evidence for parallelism in the underlying genetic basis of migratory life history divergence in this study, a few focal genome regions harbored a number of candidates for adaptive divergence. In particular, a number of markers on both *O. nerka* linkage groups 12 and 20 showed signatures of divergent natural selection, and associations with migration vs. residency in Redfish Lake. The Alturas collections also had a number of significant loci on linkage group 12. Linkage group 20 is syntenic with regions of chromosomes 16 and 20 in Rainbow Trout; all markers showing signatures of divergent natural selection in the Redfish Lake pair are syntenic with regions on chromosome 16. *O. nerka* linkage group 12 is syntenic with regions of chromosomes 9 and 29 in Rainbow Trout; all outlier markers that are aligned to the Rainbow Trout genome are found in regions syntenic only with chromosome 29. Ordering these markers along *O. nerka* linkage groups 12 and 20 would provide some insight into whether or not these loci are located in close proximity to each other; however, accurate ordering of these linkage groups is a difficult task, as there are few markers in common across the two linkage maps, which provide the framework for ordering scaffolds aligned from other species. Generally, markers showing outlier behavior on these two *O. nerka* chromosomes show fixation or reduced heterozygosity in the Redfish—Anadromous population when compared to the Redfish—Resident population (Fig. S1). The pattern could be due to the bottleneck experienced in the Redfish—Anadromous population in the early 1990s, or due to selection between life histories. An improved framework for marker order and improved coverage of the genome is necessary to provide a comprehensive picture of the possible functional significance of genes in this or other regions, or in the demographic and evolutionary processes that have shaped these differences.

### 4.2 Contrasting patterns of population differentiation

This study increased the numbers of available markers to inform analyses of population genetic structure within and between two lake systems in the upper Salmon River Basin. Both the neutral and adaptive SNPs from this study support previous allozyme studies (e.g., Waples et al. (2011)) that Redfish Lake Sockeye Salmon (Redfish—Anadromous) are genetically distinct from resident Kokanee that spawn in Fishhook Creek (Redfish—Resident), which are in turn genetically different from the resident and anadromous life history forms from Alturas Lake. The Alturas Lake fish are also more genetically similar to the Redfish—Resident (Fishhook Creek) population, than the Redfish Lake Sockeye Salmon (also previously documented by Waples et al. (2011)), despite having some propensity for anadromy. As the Redfish Lake population likely experienced a population bottleneck in the early 1990s, this may have increased levels of divergence of this population relative to others due to founder effects, although current estimates of effective population size indicate that the population has increased in size due to the captive broodstock program (this study and Kalinowski et al. (2012)). Introgression with non‐native, stocked Kokanee, known to be introduced from unknown sources (Bowler, 1990), could also lead to increased divergence among populations, but Waples et al. (2011) indicated no evidence of introgression with stocked Kokanee.

In this study, we further show that the two life history types in Alturas Lake are genetically indistinguishable on the basis of thousands of putatively neutral SNPs throughout the genome. This is not surprising, as Alturas—Anadromous individuals were assigned to the Alturas population using neutral microsatellite loci to delineate the origins of anadromous individuals in this study. However, genomic information can sometimes uncover population differentiation not seen with low numbers of neutral genetic markers (Ackerman, Habicht, & Seeb, 2011). Herein, very few markers show unusually high divergence between life history types. The differentiation between the anadromous and resident forms in Alturas Lake has beguiled scientists as Evermann noted “big” and “small” redfish in Alturas Lake in the 1890s (Evermann, 1897; Waples, Johnson, & Jones, 1991). There are two plausible historical scenarios for *O. nerka* in Alturas Lake: either that the resident and anadromous types have been maintained within the population and both were early spawners, or that these two life histories were historically distinct (early August spawner and later‐timed October spawner) but the later‐timed, anadromous migratory type has been extirpated. Evermann (1897) surveyed Alturas Lake Creek in August and observed larger fish spawning on gravel bars in the inlet and lower reach of Alturas Lake Creek, while the smaller fish spawned upstream in Alturas Lake Creek, but no surveys were conducted later in the season when October spawners would be present. Historical samples collected prior to the operation of the Sunbeam Dam may have been useful in understanding whether a genetically divergent, now‐extirpated population existed in Alturas Lake, in a manner similar to that discovered in *O. nerka* from the upper Columbia River (Iwamoto, Myers, & Gustafson, 2012). While we cannot infer what may have been historically present in the lake, the very low level of differentiation between the current resident and migratory collections in Alturas Lake supports the idea that the resident Kokanee can produce small numbers of anadromous individuals and that these individuals are capable of undertaking a successful ocean and return migration for spawning. Even in the face of strong selection against anadromy for multiple generations in Alturas Lake, there still appears to be a small number of outmigrants that survive to make the long return migration.

### 4.3 Conservation implications and applications

In species with diverse life history strategies, a conservation priority is to maintain adequate variation in order for multiple life histories to be exhibited as necessary to avoid regional extirpation of the species. Recovery strategies for endangered upper Salmon River Sockeye Salmon include the conservation of spatial population genetic and life history diversity, and protection and conservation of natural ecological processes that support the viability of populations (National Marine Fisheries Service 2015). Maintenance of life history variation in *O. nerka* populations in the upper Salmon River Basin is expected to allow for long‐term persistence by exploiting trade‐offs between resident and anadromous migration strategies (e.g., Hendry et al. (2004)). The presence of a residual life history in Redfish Lake may have been one means protecting the Redfish Lake population from going extinct during the time Sunbeam Dam (1910‐1934), a long‐time barrier to upstream migration, was in operation (Waples et al., 1991). The founders for the captive broodstock program for Redfish Lake Sockeye Salmon were collected in the years 1991–1998 and originated from a variety of life‐stages, including outmigrating smolts that were likely produced from both residual *O. nerka*, residual adult *O. nerka*, and returning anadromous Sockeye Salmon (Kalinowski et al., 2012; National Marine Fisheries Service 2015). Propagation of anadromous Sockeye Salmon in Redfish Lake has continued through the captive broodstock program, and future recovery strategies include restoring self‐sustaining, anadromous runs in Pettit and Alturas Lakes.

Information using neutral and adaptive diversity can be used to inform supplementation programs where the goals are to re‐introduce salmon or other species into their historical, natal range. Prior attempts at re‐introducing Sockeye Salmon and Kokanee throughout their range have not been successful, potentially due to the failure to capture and match the adaptive potential of the donor stock to the available spawning and rearing habitats (Burger, Scribner, Spearman, Swanton, & Campton, 2000). The present study suggests that the extremely small number of anadromous Sockeye Salmon returning to Alturas Lake since 1991 (20 fish) were produced by the resident Kokanee in that lake. The finding that resident populations of salmonids can produce migratory offspring that are successful in returning as adults is not new (Courter et al., 2013; Van Doornik, Berejikian, & Campbell, 2013), but the degree to which resident populations contribute to protected anadromous populations is an important consideration in conservation and management policies. Previous research has shown that Kokanee maintain the propensity for smoltification and can migrate to the sea and return to spawn in freshwater (Foerster, 1968; Godbout et al., 2011). While lower smolt to adult returns have been observed when compared to sockeye populations (Godbout et al., 2011), the fact that Kokanee populations harbor the ability to produce migratory offspring is an important consideration for conservation. The extent to which resident Kokanee populations can successfully lead to the establishment of consistent and abundant anadromous Sockeye Salmon populations is largely unknown, although some attempts have been made to re‐anadromize Kokanee throughout their range (Bocking & Gaboury, 2003; Foerster, 1947). Recovery options could include multiple scenarios to restore anadromous returns to Alturas Lake (summarized more fully in the 2015 ESA Recovery Plan for Snake River Sockeye Salmon; National Marine Fisheries Service (2015)). Restoration of the anadromous run in Alturas Lake from the existing resident population is one such consideration. This could include the development of a captive broodstock from outmigrating smolts produced from the Kokanee in Alturas Lake and any returning adult anadromous Alturas Lake *O. nerka*, in a manner similar to the captive broodstock development in Redfish Lake, or allowing natural reproduction of returning anadromous Alturas Lake *O. nerka* in the lake. However, it remains uncertain whether extant, resident *O. nerka* in Alturas Lake retain sufficient variation to successfully restore anadromous fish to this system, especially given that only 20 ocean‐returning adults have been discovered over the last 25 years. The introduction of anadromous fish from nearby lakes (e.g., Redfish Lake) is another consideration. On the one hand, the Redfish Lake Sockeye Salmon captive broodstock has proven effective in re‐establishing some natural reproduction of anadromous sockeye in Redfish Lake, but it cannot be known prior to re‐introduction efforts if Redfish Lake Sockeye would be adapted to survive to produce a self‐sustaining natural anadromous population in Alturas Lake. As it is uncertain which donor stock would be most successful in re‐establishing an anadromous population in Alturas Lake, any active efforts to restore endangered Snake River Sockeye Salmon may require multiple strategies; moreover, restoration efforts will require future genetic and population monitoring to understand the long‐term viability and impacts of enhanced or re‐introduced *O. nerka* on native Kokanee in those lakes (Anderson et al., 2014; McClure et al., 2008).

## 5 Conclusions

This study highlights the utility and limitations of using genomic information in restoration, conservation, and management frameworks. At the most basic level, genomic information gives an efficient, comprehensive overview of how demographic processes have shaped extant genetic variation, and provides the basis for delineating units for recovery, conservation and management, not unlike any other genetic marker used before the advent of next‐generation sequencing. Herein, our results support the delineation of three distinct populations within these two lake systems. Understanding the genetic underpinnings for divergent life history traits also contributes to our understanding of the unique, adaptive features of populations that are fundamental in developing conservation and management strategies. This study shows, at least with the available markers and sampled populations, that parallelism in migratory life history diversity is not necessarily reflected by parallel patterns of genetic differentiation. Drawing upon conclusions herein, and from other studies demonstrating the lack of parallelism, caution should be used in making conservation decisions for specific management units on the basis of observations in other species or populations. Understanding the genetic underpinnings of ecological life history divergence is complicated not only by complex evolutionary scenarios across heterogeneous landscapes, but also by complex demographic and ecological histories shaped by anthropogenic impacts. Although many studies to date fail to provide a comprehensive picture of how parallel phenotypic diversity is shaped by underlying genetic diversity, more complete genome resources are obtainable in virtually any species, and will be imperative in better understanding how extant genetic and life history diversity in plants and animals of conservation concern have been shaped over evolutionary and ecological time scales. While genomic studies may be able to provide increased resolution for understanding adaptive diversity, until correlations between molecular genetic and adaptive phenotypic diversity can be made in species and populations of concern, neutral genetic diversity and phenotypic diversity will continue to be central metrics for decision‐making processes in restoration, conservation, and management strategies.

## Data Sharing/Archiving

Raw sequence data for this study are available at the NCBI Sequence Read Archive, accession PRJNA336345. Sequencing barcodes and metadata are provided in Table S1. Genotypes used for analyses are provided in Appendix S2.
