# Supplementary material for: Genomic signatures among Oncorhynchus nerka ecotypes to inform conservation and management of endangered Sockeye Salmon
Source: Evol Appl. 2016 Oct 21;9(10):1285–300. doi: 10.1111/eva.12412 (PMC5108219; doi:10.1111/eva.12412)

Figure S1. Boxplots for observed heterozygosity calculated for RAD loci localized to *O. nerka* linkage groups 12 (A) and 20 (B) in the Redfish - Anadromous (RFL-A) and Redfish - Resident (RFL-R) populations. Boxplots are presented for those that were ('yes' on x-axis) or were not ('no' on x-axis) outlier loci.

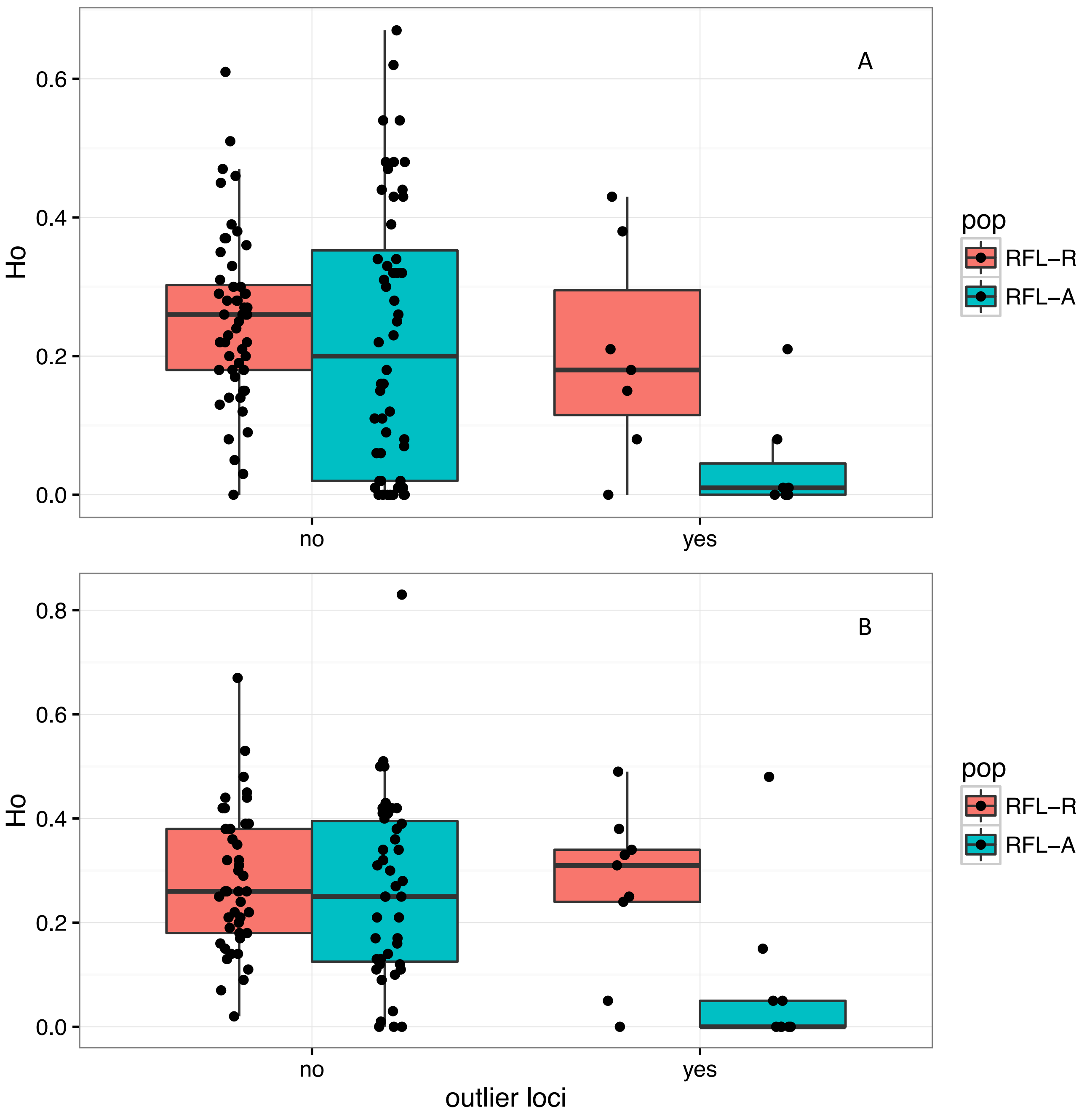

Figure S2. Mean Q plots for each individual with K=3 clusters in fastStructure. The three colors represent the membership in each of three colors, while each vertical line represents an individual in each of the three populations.

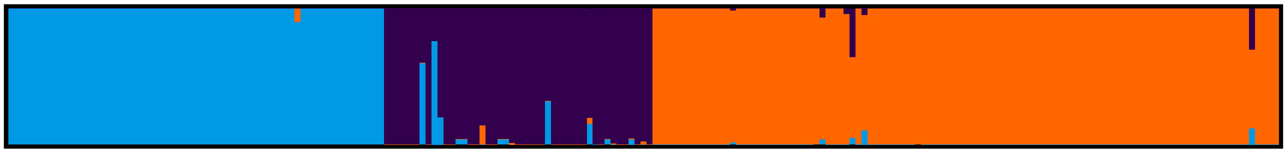

Figure S3. QQ plots from mixed linear model in GAPIT for genome-wide association analyses. Three analyses were run: 1) all collections (n = 211), where the model included PC1 and sex, 2) the Alturas collections only (n = 63), where the model included sex, and 3) the Redfish collections only (n = 148), where the model included PC1, PC2 and sex. All models included the uncompressed kinship matrix.

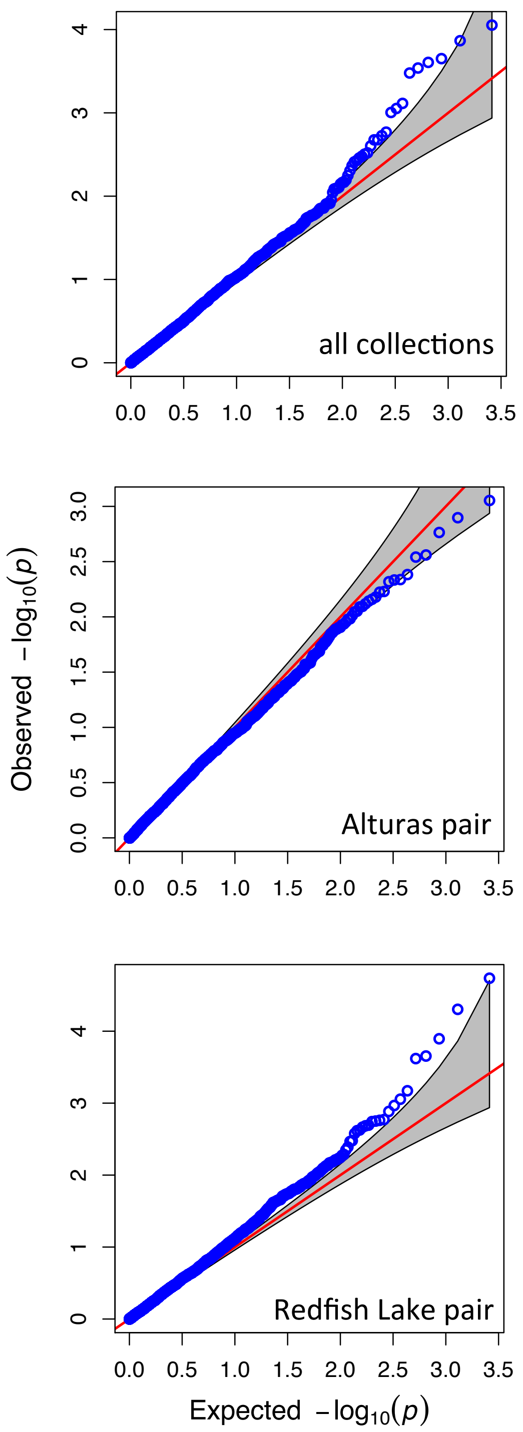

Figure S4. Proportion of variation explained (PVE) by top subsets of markers in the random forest. Top subsets are proportion of the total n = 2593 markers. The top panel shows the full range of top subsets, and the bottom panel is a zoomed snapshot of the PVE from the top panel for 0-10% top subsets.

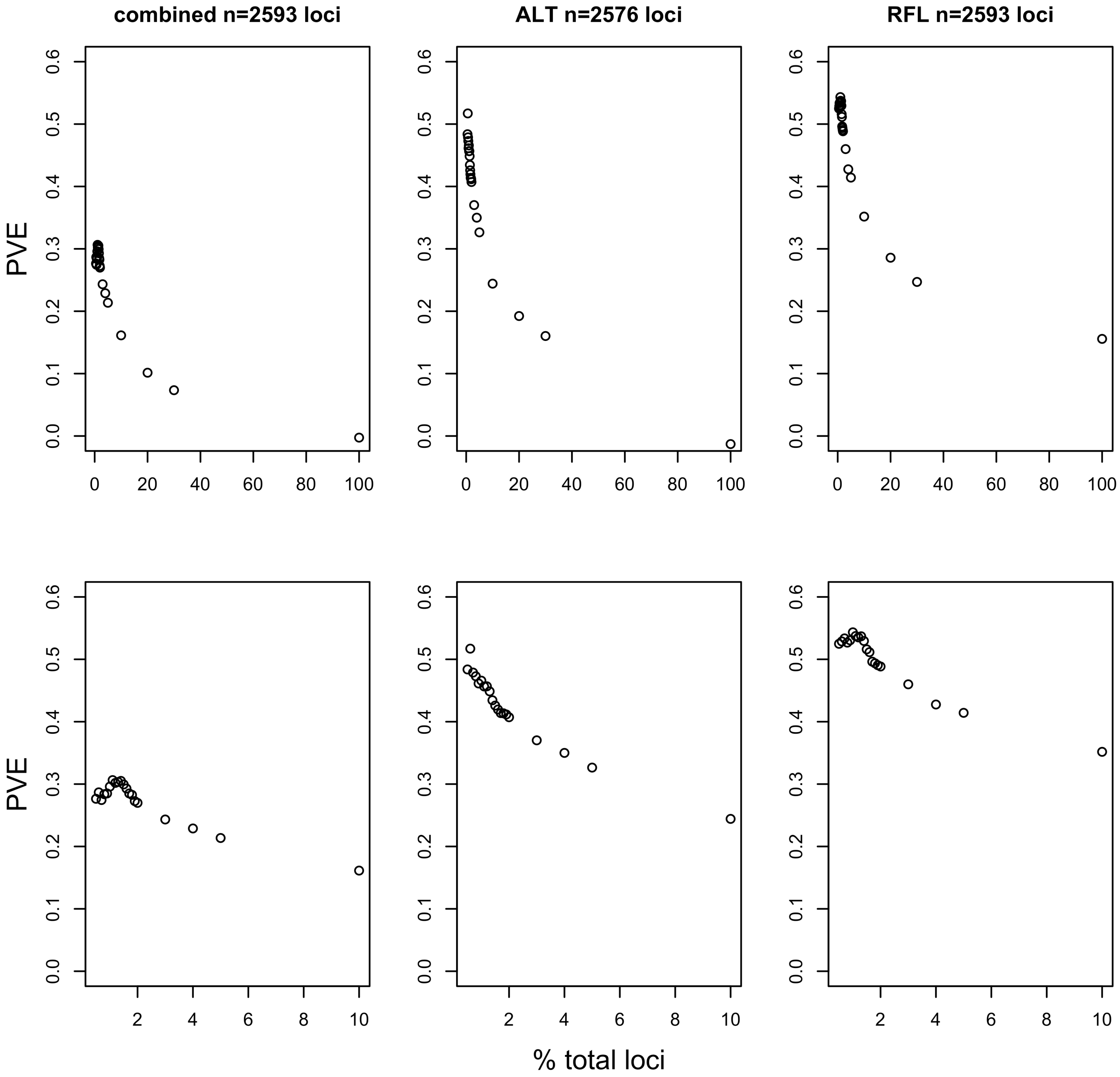

Figure S5. Average proportion of variation explained by the top 77, 28, and 66 loci in the backwards purging model selection for random forest in the combined, Alturas, and Redfish collections, respectively.

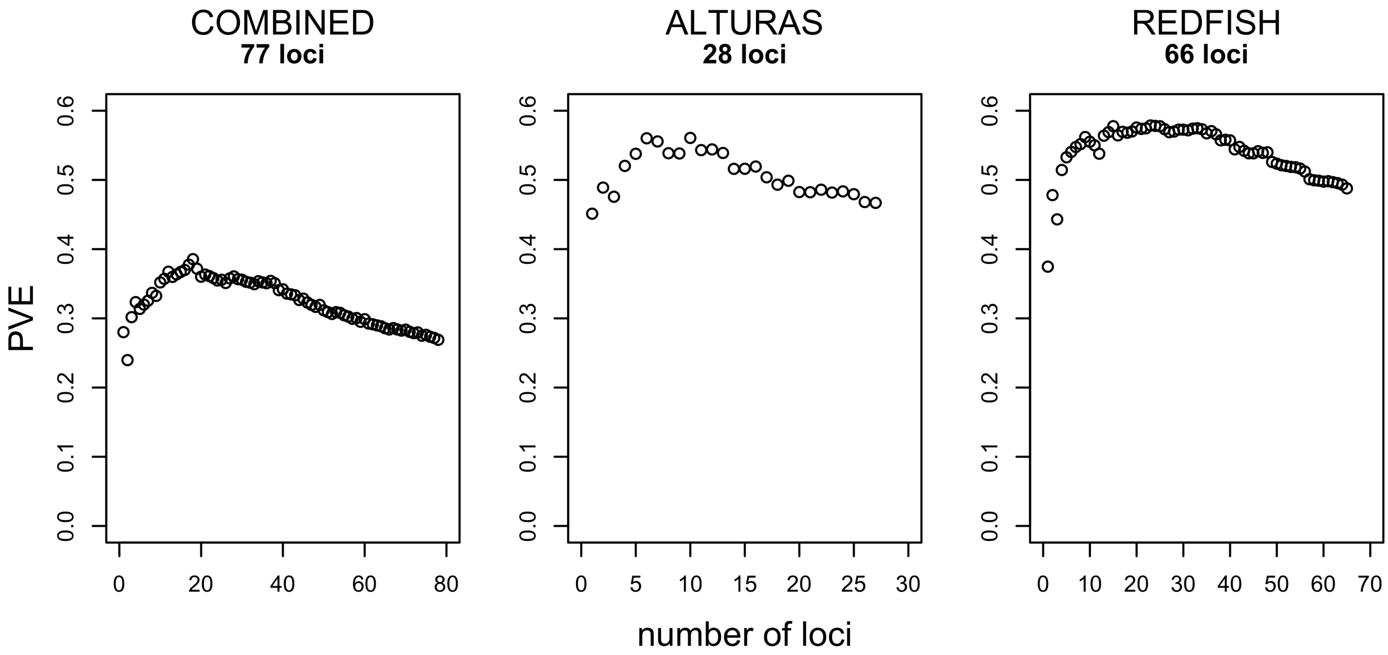

Figure S6. Heatmap of pairwise linkage disequilibrium (r^2) between all SNPs significant in at least one model tested in this study.

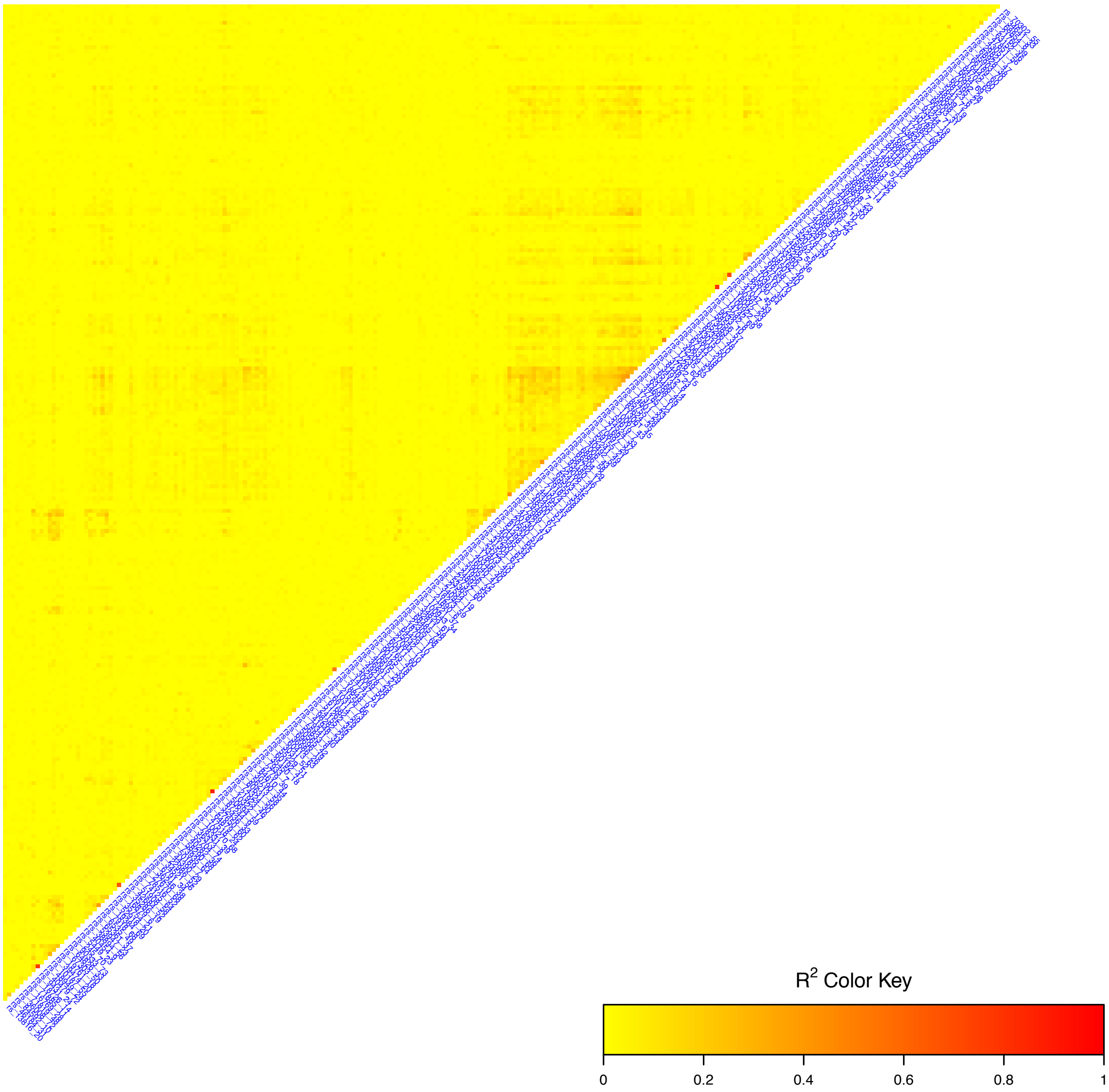

Supplement: Supplementary file 1 [file EVA-9-1285-s001.pdf]
