# Supplementary material for: Genomic signatures among Oncorhynchus nerka ecotypes to inform conservation and management of endangered Sockeye Salmon
Source: Evol Appl. 2016 Oct 21;9(10):1285–300. doi: 10.1111/eva.12412 (PMC5108219; doi:10.1111/eva.12412)
Supplement: Supplementary file 3 [file EVA-9-1285-s003.pdf]

Appendix S1: Identification of Alturas Lake Origin Returning Adults

Anadromous Adult Returns

Anadromous adults that return to the Sawtooth Valley basin are either trapped at an adult trap on Redfish Lake Creek or the Sawtooth Fish Hatchery weir on the upper Salmon River (Figure 1; Peterson et al. 2014). Upon trapping, a fin clip is collected along with information regarding gender, length, weight and mark information. For this study, DNA was extracted using a Nexttec DNA isolation kit according to the manufacturer's instructions (http://www.nexttec.biz). Following DNA extraction, each sample was amplified with 13 microsatellite loci: One103, One104, One108, One111, One112, One114, One115, Oki1, One13, One110, One106, Omy77, and Ots103. (Scribner et al. 1996; Smith et al. 1998; Beacham et al. 1998; Cairney et al. 2000; Olsen et al. 2000; Perry et al. 2001; Rexroad et al. 2001). Multiplex reactions were carried out for four combinations of loci (contact the authors for PCR concentrations and thermocycler profiles). Following amplification, PCR fragments were diluted at 1:30 ratio and size fractionated using an ABI3730 capillary DNA sequencer and internal size standard (GS500) genotypes were scored by GeneMapper software version 3.0 (Applied Biosystems) Individuals representing ~10% of the sampled fish were re-amplified and scored a second time. After each anadromous fish was genotyped with a minimum of 9 loci, individuals with alleles that were not found in the captive broodstock were flagged.

Reference Baseline

Genetic assignment tests were used to assign anadromous fish back to the baseline. For this analysis, the reference baseline included 4,072 *O. nerka* from the captive broodstock program and the Sawtooth Valley lakes (Table 1). This reference baseline was generated from a number of different collection

types (creel, trawl, creek spawners, captive broodstock) taken as part of research, monitoring and evaluation efforts for the Snake River Sockeye Salmon program and is updated annually.

Table 1: Collection Information including collection group, year sampled and sample size

Collection	Years	Sample Size
Fishhook Creek Kokanee	2005 - 2013	321
Warm Lake Kokanee	2003	40
Pettit Lake Kokanee	2000 - 2010	42
Deadwood Reservoir Kokanee	2007 - 2008	55
Stanley Lake Kokanee	2005, 2010	36
Alturas Lake Kokanee	2005-2013	1078
Redfish Lake Sockeye Salmon Captive Broodstock and Anadromous Returns	2000 - 2012	2500
	Total	4,072

The software Geneclass 2.0h (Piry et al. 2004) was used to determine the self-classification rate of the collections within the baseline. Both a Bayesian assignment test (Rannala and Mountain 1997) and a frequency based test (Paetkau et al. 1995) were used to confirm identity. Individual assignments to a population with greater than 90% probability were accepted. For the frequency assignment test, a frequency of 0.01 was used to adjust the allele frequencies in the baseline populations if it was present in the tested individual but not the population. Overall, high self-assignment rates were observed indicating high differentiation among populations within the baseline and the ability to correctly assign individuals back to their population of origin (Table 2).

Table 2: Self-classification assignments of the reference baseline using both the a) frequency based assignment test and b) Bayesian assignment test.

a) Frequency Test Results

Collection	Broodstock	Alturas	Deadwood	Pettit	Stanley	Warm Lake	Fishhook Creek	Not assigned	% Self Assignment
Broodstock	2495							5	99%
Alturas		869					20	189	80%
Deadwood			52					3	95%
Pettit				39				3	90%
Stanley					35			1	97%
Warm						40			100%
Fishhook Creek		2					285	34	89%

b) Bayesian Test Results

Collection	Broodstock	Alturas	Deadwood	Pettit	Stanley	Warm Lake	Fishhook Creek	Not assigned	% Self Assignment
Broodstock	2498							2	99%
Alturas		999					11	68	93%
Deadwood			55						100%
Pettit		1		39			1	1	90%
Stanley			2		34				94%
Warm						40			100%
Fishhook Creek		2					293	26	91%

Detection of Alturas Lake Individuals

Given the high self-assignment rates back to the baseline, both assignment tests were used to assign anadromous adults back to the baseline *O. nerka*. As stated above, anadromous fish returning from 2008 – 2010 were genotyped and 17 fish assigned to the Alturas Lake population (Table 3). Two additional samples assigned to either the Alturas Lake population or captive broodstock program, depending on the assignment test and were not included in the subsequent analyses. The software STRUCTURE 2.0 also confirmed population membership of these same anadromous individuals to the kokanee population when using the same reference baseline and forcing the program to allocate

individuals to two clusters (e.g. captive broodstock or Sawtooth Valley Kokanee; data not shown). The FASTSTRUCTURE analysis using the SNP data also confirmed population membership of those individuals retained for population genomic and association analyses (see Figure S1).

Table 3: Individuals that assigned to the Alturas Lake population

Sample	Probability with Frequency Test	Probability with Bayesian Test
OneEAGL08AAN0001	99.957	99.951
OneEAGL09AAN0038	97.28	99.990
OneEAGL09AAN0454	84.885	98.118
OneEAGL10AAN0025*	99.989	100.00
OneEAGL10AAN0097	99.636	100.00
OneEAGL10AAN0122	99.991	100.00
OneEAGL10AAN0146*	97.548	99.991
OneEAGL10AAN0159	81.253	99.999
OneEAGL10AAN0202	99.885	100
OneEAGL10AAN0235	70.861	99.747
OneEAGL10AAN0270	97.251	100
OneEAGL10AAN0281	79.079	99.815
OneEAGL10AAN0367	96.844	96.895
OneEAGLE10AAN463	99.965 (FH)	78.877 (FH)
OneEAGL10AAN0524	99.697	99.700
OneEAGL10AAN0571	99.993	100
OneEAGL10AAN0582	67.603	67.996
OneEAGL10AAN0608	83.131 (FH)	98.665
OneEAGL10AAN0665	99.963	100

*Two fish that were removed from the RAD sequencing analysis because they were missing >25% genotypes in the filtered SNP set and two fish were removed due to ambiguous assignment as assignment to the Fishhook Creek (FH) resident population

References:

Beacham, T. D., L. Margolis and R. J. Nelson. 1998. A comparison of methods of stock identification for sockeye salmon (*Oncorhynchus nerka*) in Barkley Sound, British Columbia, North Pacific Anadromous Fish Commission, Bulletin No. 1: 227-239.

Cairney, M., J.B. Taggart and B. Hoyheim. 2000. Characterization of microsatellite and minisatellite loci in Atlantic salmon (*Salmo salar* L.) and cross-species amplification in other salmonids. Molecular Ecology 9: 2175-2178.

Olsen, J. B., S. L. Wilson, E. J. Kretschmer, K. C. Jones and J. E. Seeb. 2000. Characterization of 14 tetranucleotide microsatellite loci derived from sockeye salmon. Molecular Ecology 9: 2185-2187

Paetkau, D., W. Calvert, I. Stirling and C. Strobeck. 1995. Microsatellite analysis of population structure in Canadian polar bears. Molecular Ecology 4: 347-354.

Perry, G. M. L., G. J. McDonalds, M. M. Ferguson, R. C. Ganassin and N. C. Bols. 2001. Characterization of rainbow trout cell lines using microsatellite DNA profiling. Cytotechnology 37: 143-151.

Piry, S., A. Alapetite, J.-M. Cornuet, D. Paetkau, L. Baudouin and A. Estoup. 2004. GeneClass2: A Software for Genetic Assignment and First-Generation Migrant Detection. Journal of Heredity 95: 536-539.

Rannala, B. and J. L. Mountain. 1997. Detecting immigration by using multilocus genotypes. Proceedings of the National Academy of Sciences 94: 9197-9201.

Rexroad, C. E., R. L. Coleman, A. M. Martin, W. K. Hershberger and J. Killefer. 2001. Thirty-five polymorphic microsatellite markers for rainbow trout (*Oncorhynchus mykiss*). Animal Genetics 32: 317-319.

Scribner, K. T., J. R. Gust and R. L. Fields. 1996. Isolation and characterization of novel salmon microsatellite loci: cross-species amplification and population genetic applications. Canadian Journal of Fisheries and Aquatic Sciences 53: 833-841.

Smith, C. T., B. F. Koop and R. J. Nelson. 1998. Isolation and characterization of coho salmon (*Oncorhynchus kisutch*) microsatellites and their use in other salmonids. Molecular Ecology 7: 1614-1616.
